# Multimorbidity and animal models

**DOI:** 10.1002/ame2.70119

**Published:** 2025-12-22

**Authors:** Xinpei Wang, Yakun Ren, Xingjiu Yang, Mengyuan Li, Junxiu Liu, Xiaoyan Du, Wen Wang, Ran Gao

**Affiliations:** ^1^ State Key Laboratory of Respiratory Health and Multimorbidity & Institute of Laboratory Animal Science CAMS & PUMC Beijing China; ^2^ National Human Diseases Animal Model Resource Center, National Center of Technology Innovation for Animal Model, Institute of Laboratory Animal Science CAMS & PUMC Beijing China; ^3^ NHC Key Laboratory of Comparative Medicine CAMS & PUMC Beijing China; ^4^ Department of Population Health Science and Policy Icahn School of Medicine at Mount Sinai New York New York USA; ^5^ The Department of Laboratory Animal Science, School of Basic Medical Science Capital Medical University Beijing China

**Keywords:** animal models, biological complexity, multimorbidity

## Abstract

Multimorbidity—the co‐occurrence of more than two chronic conditions in the same individual—is associated with premature death, diminished function, reduced quality of life, and increased societal burden. This complex state involves dynamic interactions across multiple conditions, organ systems, and physiological pathways; yet research progress remains constrained by inadequate animal models that recapitulate human complexity. This review summarizes the predominant patterns of multimorbidity and evaluates current animal models spanning invertebrates, rodents, and large mammals. While no single model fully captures the multifaceted nature of human multimorbidity, we propose several strategic directions to address existing limitations: implementing a cross‐species validation framework (from simple organisms to rodents to large mammals), standardizing protocols integrating multimodal risk factors, developing advanced non‐animal models, and enhancing ethical oversight. Advancing multimorbidity models is crucial for decoding disease interactions and accelerating translation of research findings into improved patients outcomes.

## Graphical abstract

Multimorbidity, defined as the coexistence of ≥2 chronic conditions, is associated with aging, genetics, and environmental factors. Animal models in multimorbidity research span three tiers: simple organisms for initial screening → rodents for mechanistic analysis → large mammals for clinical prediction.

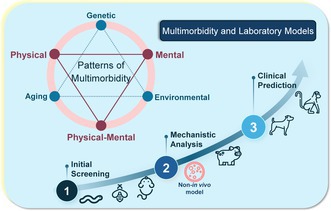

## 1 INTRODUCTION

**FIGURE 1:**
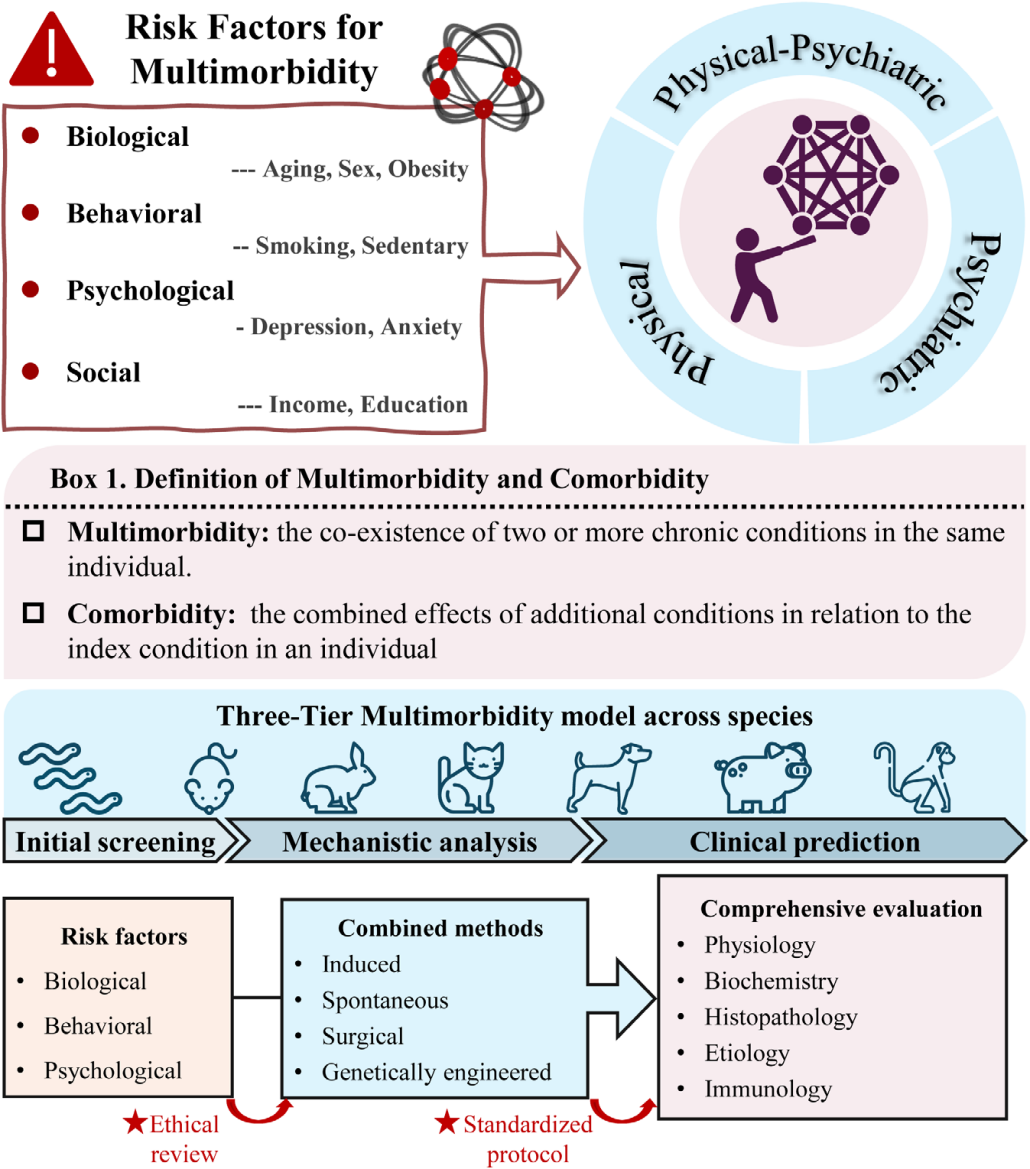
Multimorbidity and laboratory animal models for multimorbidity research. This figure outlines major risk categories (biological, behavioral, psychological, and social) associated with multimorbidity and clearly defines the core concepts of “multimorbidity” and “comorbidity”. Guided by ethical review and standardized protocols, a three‐tier model for multimorbidity research across species is presented, spanning initial screening, mechanistic analysis, and clinical prediction, incorporating various methodological and evaluative approaches, all guided by ethical review and standardized protocols.

Multimorbidity, defined as the coexistence of two or more chronic conditions in an individual, represents a substantial global challenge affecting individuals, caregivers, and society.^1^ It is essential to distinguish multimorbidity from comorbidity (Figure 1). Comorbidity specifically refers to additional conditions occurring in relation to an index disease, whereas multimorbidity describes the co‐occurrence of multiple conditions without presumed causality.^2^, ^3^ The prevalence and severity of multimorbidity vary across countries, races, genders, and ages, with generally higher rates observed in high‐income countries, among females, and in individuals aged over 60.^4^ The rising burden of multimorbidity is driven by a complicated network of risk factors, including biological determinants (aging, inflammation), social factors, behavioral issues, psychosocial elements, and prior infections (Figure 1).^5^ Figure 1 summarizes this complex network, highlighting the challenge of modeling such multifactorial causality in laboratory animal models.

**FIGURE 2:**
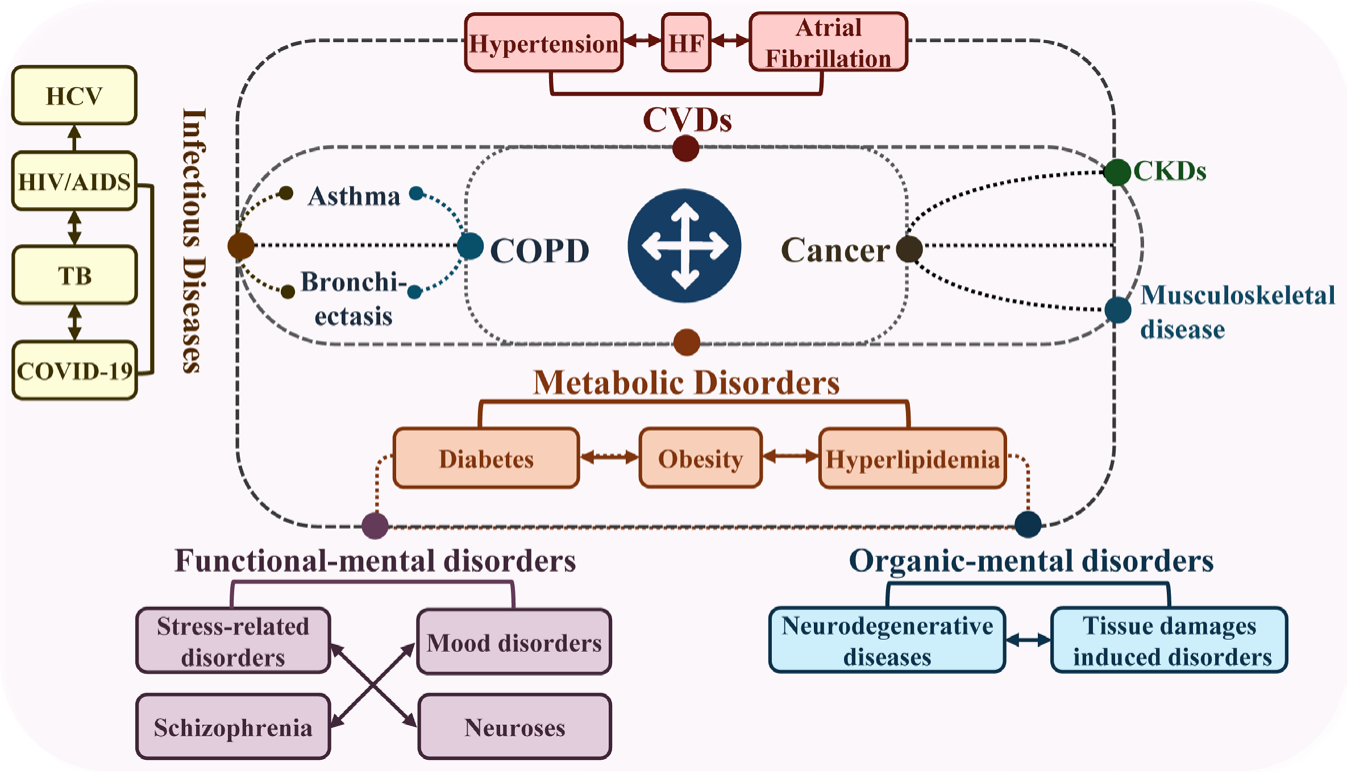
The main patterns of multimorbidity. This figure presents the broad spectrum of conditions in multimorbidity, categorized into two principal domains: Somatic diseases (including cardiovascular, metabolic, respiratory, cancers, and infections) and neuropsychiatric disorders (both functional and organic). It illustrates the bidirectional relationships and frequent co‐occurrence among disorders within and between these domains (physical–physical, psychiatric‐psychiatric, and physical‐psychiatric). This interplay forms an interconnected disease network that promotes multimorbidity and worsens disease progression.

Despite its clinical importance, establishing causality mechanisms for multimorbidity has proven been profoundly challenging. Current models often focus on single diseases, neglecting the complexity inherent in multimorbidity.^6^ A fundamental challenge lies not only in modeling multiple conditions simultaneously but also in capturing the dynamic, often non‐linear interactions between them over a relevant timescale, which many current models fail to achieve. This review outlines the main multimorbidity patterns (Figure 2), evaluates existing animal models for multimorbidity research (Table 1), and discusses future directions for developing more predictive and pathophysiological animal models to better understand disease interactions and advance therapeutic interventions.

## 2 METHODOLOGY

This narrative review synthesizes current knowledge regarding multimorbidity patterns and the related animal models. Literature searches were performed in PubMed and Web of Science Core Collection, primarily covering 2020 to September 2024, and supplemented by searches from 2010 to 2019. The search strategy combined keywords and MeSH terms for three concepts: (“multimorbidity” OR “multi‐morbidity” OR “comorbidity”) AND (“animal model” OR “Caenorhabditis elegans” OR “mouse” OR “rat” OR “Golden Syrian hamster” OR “dog” OR “porcine” OR “pig” OR “swine” OR “non‐human primate” OR “NHP”) AND (“multiple conditions”). Inclusion criteria were: (1) experimental animal studies involving two or more concurrent or sequentially developed chronic diseases; (2) studies addressing interactions or shared mechanisms between the conditions; (3) original research articles or reviews published in English. Studies focusing on a single index disease with acute complications or lacking in vivo data were excluded. The review is structured to progress from a descriptive summary of major multimorbidity patterns (physical, psychiatric, physical‐psychiatric) to a critical analysis of models across species.

## 3 MAJOR PATTERNS OF MULTIMORBIDITY

### 3.1 Physical multimorbidity

Physical multimorbidity, the coexistence of multiple physical health conditions in an individual, is particularly common among older adults.^7^ It can be broadly divided into two clusters:

#### 3.1.1 Non‐communicable disease (NCD) multimorbidity

This clusters refers to individuals with an initial NCD, including cardiovascular disease (CVD), cancers, diabetes, and chronic respiratory disease (CRD), with the development of one or more subsequent NCDs.^8^

##### Cardiovascular disease (CVD)

CVD is frequently at the center of multimorbidity clusters (Figure 2), and there is an alarming global prevalence of CVD‐associated multimorbidity. In the UK, over 24.3% of individuals have five or more conditions, including hypertension, depression, arthritis, asthma, and anxiety, while in China, approximately half or more of CVD patients present with concurrent conditions such as hyperlipidemia, diabetes, and hypertension.^9^, ^10^ The mechanisms are multifactorial, involving cardiovascular aging, sex hormones, sex chromosomes, lifestyle, and genetic factors.^11^ However, the precise temporal sequence and causal hierarchy among these factors remain unclear, demanding animal models capable of decoupling these intertwined processes through longitudinal studies.

##### Type 2 diabetes (T2D)

T2D patients commonly experience multiple co‐occurring conditions, with hypertension, musculoskeletal disease, and hyperlipidemia being the most common pattern.^12^, ^13^ Insulin resistance, chronic inflammation, oxidative stress, hormonal imbalances, epigenetic changes, and medications like metformin are linked to diabetes‐related multimorbidity.^14^, ^15^ Nevertheless, the molecular triggers that propel the transition from a single disease to a multimorbid state remain poorly defined. This underscores the need for models that explore how pre‐existing metabolic dysfunction alters the trajectory of secondary conditions.

##### Cancers

Despite improved cancer survival rates, multimorbidity among cancer survivors is rising globally.^16^ The most prevalent patterns involve diabetes, hypertension, arthritis, pulmonary disease, stroke, and angina or coronary heart disease.^17^ The reasons are multifaceted, with aging serving as a central risk factor. Chronic infections, compromising immune function, metabolic dysfunction, and long‐term medication use, also contribute.^18^, ^19^, ^20^ There is a pressing need for animal models that can control for confounding variables to isolate these biological interactions. These models are crucial to determine whether a multimorbid state like diabetes directly promotes cancer progression beyond shared etiological factors.

##### Chronic obstructive pulmonary disease (COPD)

COPD is now the third leading cause of death worldwide.^21^ Beyond primary impairment of lung function, more than 90% of COPD patients have at least one chronic condition, while over 50% have three or more.^22^ COPD‐related multimorbidities include both pulmonary and extrapulmonary diseases, notably CVD, metabolic disorders, psychiatric and neurological disorders, with most COPD patients ultimately dying from non‐respiratory causes.^23^ The pathological connections between COPD and other diseases involve aging, genetic susceptibility, and systemic inflammation. The “spillover” of pulmonary inflammation is a leading hypothesis.^24^ Nonetheless, the specific mediators linking lung damage to remote organ dysfunction remain elusive, calling for models that replicate systemic consequences.

#### 3.1.2 Communicable disease multimorbidity

This pattern refers to individuals having at least one infectious disease and one or more communicable/non‐communicable disease.^25^

##### HIV

HIV primarily targets immune cells, resulting in impaired immune function and higher prevalence of mental disorders, metabolic disorders, CVD, cancers, and infections.^26^ Exploring the contribution of chronic immune activation, aging, lifestyle, and antiretroviral therapy (ART) to these multimorbidities is challenging, primarily due to the limitations of current animal models. Conventional rodents are resistant to HIV‐1, while humanized mice often lack a fully functional immune system.^27^ Simian Immunodeficiency Virus (SIV)‐infected non‐human primates recapitulate key aspects of pathogenesis, but they are costly and fail to model the impact of ART.^28^ Consequently, novel models are needed that not only permit viral replication but also capture the chronic systemic sequelae of HIV.

##### TB

TB is the second leading cause of death from a single infectious disease globally, and multimorbidity involving TB is common.^29^ The relationship between TB and chronic physical conditions is often bidirectional.^30^ TB is also a leading cause of death among people with HIV, with an estimated 167 000 deaths from HIV‐associated TB.^29^ Macrophages are regarded as potential cellular targets that link HIV and TB infections, but co‐infection of the two pathogens within macrophages has not been observed. This complex host‐pathogen interaction highlights the need for co‐infected models to elucidate the underlying molecular mechanisms.

##### COVID‐19

COVID 19 was initially characterized as an acute respiratory illness, but has lasting effects on multiple systems. The combination of CKD and diabetes was associated with the highest risk of severe COVID‐19.^31^ The relationship between COVID‐19 and diabetes is complex: pre‐existing diabetes can worsen COVID‐19 outcomes due to chronic inflammation and endothelial dysfunction, while COVID‐19 can also trigger new‐onset diabetes, possibly due to lasting β‐cell damage.^32^ Identifying causal relationships between multiple conditions and outcomes is methodologically challenging, reinforcing the value of controlled animal models to isolate and validate specific mechanistic pathways.

### 3.2 Psychiatric multimorbidity

Psychiatric multimorbidity refers to the coexistence of three or more psychiatric disorders in the same individual, significantly contributing to the global disease burden.^33^ As shown in Figure 2, psychiatric disorders are classified as organic (linked to brain damage or pathological changes) and functional (without such physical evidence). The presence of a single mental disorder often raises the risk of developing others.^34^ Age is a key risk factor, with depression and dementia mutually increasing each other's risks in older adults.^35^ This link may be mediated by abnormal neurogenic activity, fewer hippocampal granule neurons and poor neuronal maturation.^36^ Beyond psychiatric conditions, chronic pain also contributes to neurological morbidity, as it is associated with accelerated cognitive decline, and sometimes can even present as a premotor symptom for Parkinson's disease (PD).^37^, ^38^

Research on psychiatric multimorbidities encounters specific challenges. Rodent models often fail to fully replicate human advanced emotional and behavioral traits due to differences in cell types, gene expression profiles, and developmental trajectories, creating major gaps in studies of psychiatric multimorbidities. Recent human genetics research has identified numerous risk‐increasing allelic variants, indicating the polygenic nature of psychiatric disorders, but the molecular links between these conditions remain largely unknown.^39^ This represents a fundamental gap that current animal models, often focused on single‐disorder endpoints, are ill‐equipped to address.

### 3.3 Physical‐psychiatric multimorbidity

This is defined as the co‐occurrence of at least one physical illness and at least one psychiatric disorder.^40^ There is a bidirectional relationship between physical and psychiatric conditions.^41^ Among people with mental disorders, CVD is the most common cause of death for both genders, followed by cancer and respiratory diseases.^42^

#### 3.3.1 Mental disorders and CVD

Mental disorders and CVD are linked through several pathways like heightened oxidative stress, increased heart rate, and elevated inflammatory markers. Individuals with mental disorders exhibit increased cardiovascular risk and mortality across all age groups.^43^ Conversely, CVD also increases susceptibility to mental disorders with shared pathophysiological features, including metabolic, immuno‐inflammatory, autonomic, and hypothalamic–pituitary–adrenal (HPA) axis dysregulation.^44^, ^45^ Yet the precise mediators of this bidirectional link remain unclear, requiring models that capture this two‐way relationship to move beyond correlation.

#### 3.3.2 Mental disorders and cancer

Mental disorders and cancer show a complex interplay. Mental disorders are known to increase cancer mortality, but their impact on incidence is less clear, varying by cancer type and specific mental disorder. Studies show depression and anxiety increase risks of certain cancers and cancer‐specific mortality, while schizophrenia and bipolar disorder are linked to higher overall cancer incidence, particularly lung cancers.^46^, ^47^ These clinical findings underscore a complex, bidirectional relationship between specific mental disorders and cancer. However, current animal models often lack a comorbid dimension, leaving mechanisms such as how chronic stress and/or neuro‐inflammation drive organ‐specific tumor development largely unaddressed.

#### 3.3.3 Diabetes and depression

Diabetes and depression are closely linked via a bidirectional relationship. In depression, increased release and activity of counter‐regulatory hormones, altered glucose transportation, and heightened immunoinflammatory activation collectively raise T2D risk.^48^ In diabetes, long‐term antipsychotic use in T2D patients, associated with side effects like obesity and metabolic abnormalities, increases depression risk.^49^ Understanding how the co‐occurrence of diabetes and depression worsens metabolic control, increases vascular complications, accelerates cognitive impairment, and raises overall mortality is clinically crucial. Therefore, the development of sensitive animal models that mimic this bidirectional pathophysiology is critically needed for preclinical research and therapeutic testing.

## 4 ANIMAL MODELS FOR MULTIMORBIDITY

Animal models are crucial for understanding human diseases and developing medicines, vaccines, and therapies. However, frequently used animal models often fail to replicate the complexity of human diseases, particularly in the context of multimorbidities. This review summarizes the efforts to model multimorbidity across different species, as shown in Table 1.

### 4.1 *Caenorhabditis elegans* (*C. elegans*)

*C. elegans* serves as a powerful tool for initial discovery in age‐related multimorbidities, due to its short lifespan, genetic tractability, and optical transparency. Over 100 aging‐related obesity‐linked genes have been identified in *C. elegans*, many conserved in mammals, offering insights into the aging‐obesity link.^50^ Beyond host genetics, Wan et al. discovered a transgenerational epigenetic signal in *C. elegans* potentially transmit unhealthy aging markers across generations.^51^ In neurodegenerative disease studies, transgenic *C. elegans* expressing human pathological proteins such as beta‐amyloid (Aβ) and tau have been developed for AD research, with their transparent bodies facilitating real‐time, in vivo observation of protein aggregation.^52^ Despite limitations such as the absence of complex immune responses, advanced neural functions, and complex behaviors, *C. elegans* remains a powerful model in age‐related multimorbidity research, particularly for genetic mechanisms and large‐scale drug screening.

### 4.2 Rodents

#### 4.2.1 Mice

Mice share substantial genetic similarity with humans and are used in nearly all types of human disease research. Genetically engineered mice (GEM) have boosted multimorbidity research by introducing multiple pathogenic genes into a single mouse to explore multi‐disease interactions. In COVID‐19 studies, mouse models of CVD and diabetes engineered to express human ACE2 (hACE2) have been used to study the mechanisms underlying increased severity and mortality in patients with these conditions.^53^, ^54^ In psychological multimorbidity research, Cui et al. induced depression in mice using chronic unpredictable mild stress (CUMS) and established tumor‐bearing models, revealing that depression promotes lung cancer progression by increasing PD‐L1 expression, and reducing CD8^+^ T cell levels.^55^ Beyond traditional laboratory mice, the Collaborative Cross (CC) mice, derived from eight founder strains, broaden multimorbidity research.^56^ Milhem et al. used CC mice to identify gender‐related multimorbidities of intestinal cancer, T2D, and obesity, revealing complex genetic interactions and gender‐specific susceptibilities.^57^ CC mice highlight the role of genetic diversity in understanding multimorbidities.

Though widely used for human disease modeling, mice have limitations in multimorbidity research. Species differences between mice and humans may compromise the translational validity. Additionally, mice exhibit relatively low behavioral complexity, potentially limiting their use in psychiatric multimorbidity studies. Future research will concentrate on developing mouse models that integrate multiple factors for more complicated modeling of human conditions.

#### 4.2.2 Rats

Rats offer physiological advantages over mice, particularly in cardiometabolic and neuropsychiatric research, due to their more human‐like physiology and more complex behavioral repertoire.^58^ Furthermore, rats share greater similarities with humans in immune system function, especially in T cells and macrophages, making them better suited for research on immune‐related multimorbidity.^59^ Advances in gene editing technologies have made genetic manipulation in rats more feasible, and future efforts will focus on developing more humanized rat models to bridge the gap between animal models and human conditions.

#### 4.2.3 Golden Syrian hamsters

Golden Syrian hamsters are valuable for studying infectious and cardiometabolic multibodbidity research due to unique host susceptibility. Golden hamsters support SARS‐CoV‐2 replication and exhibit clinical features similar to those observed in humans, such as age‐dependent increases in mortality and severe illness rates.^60^ In cardiometabolic‐psychiatric research, unlike common rodents, golden hamsters develop increased appetite and weight gain under stress, revealing a link between chronic stress and obesity that mirrors human responses.^61^ By leveraging their advantages in host immune recognition, cardiometabolic response, and emotional behavior, the continued development of hamster models for both physical and psychological multimorbidity will provide a unique platform for elucidating the interactions among these complex diseases.

#### 4.2.4 *Octodon degus* (degu)

The degu is from north‐central Chile, belonging to the *Octodontidae* family with an average lifespan of 5–8 years. Degus exhibit human‐like traits: complex social behaviors, a diurnal circadian pattern, and spontaneous development of age‐related disorders.^62^ Unlike standard laboratory mice and rats, degus spontaneously develop AD‐like pathology and T2D, offering a natural aging model.^63^, ^64^, ^65^ Despite their promise as a natural aging model, access to degus is limited, and standardized strains and breeding protocols are lacking. Developing a characterized laboratory strain of degus could enhance the reproducibility of research.

### 4.3 Dogs

Domestic dogs (*Canis familiaris*), which share the human environment, are ideal for studying age‐related and environment‐genetic multimorbidities. They exhibit vast phenotypic diversity in size, shape, color, behavior, expected lifespan, and disease susceptibility, paralleling human variation.^66^ Moreover, dogs undergo multisystemic functional decline with aging and develop a spectrum of age‐related conditions comparable to humans, including cancer, kidney disease, impaired cognition, sarcopenia, diabetes and obesity, eye disorders and cataracts, cardiac abnormalities, and osteoarthritis.^67^ Two pioneering efforts in dogs—the Golden Retriever Lifetime Study (GRLS) and the Dog Aging Project (DAP)—aimed to assess genetic, environmental, aging, and dietary risk factors for multimorbidity through longitudinal tracking.^68^, ^69^ However, they also highlight a central challenge in translating vast observational data into actionable, mechanistic insights for human health. In addition, the high cost of large cohorts remains a crucial limitation.

### 4.4 Pigs

Pigs exhibit striking anatomical, physiological, immunological, and genomic similarities to humans. Coupled with their shorter reproductive cycles and more accessible genetic editing technologies, pigs represent a superior platform for translational validation of complex multimorbidity clusters.^70^ Inducible pig models have been developed for cardiometabolic multimorbidities. Wouw et al.^71^ developed a multi‐condition swine model with diabetes, hypercholesterolemia, and CKD, linking their combination to heart failure (HF)‐related pulmonary vascular disease. Gerrity et al.^72^ induced diabetes in an atherosclerotic swine model to study its interaction with atherosclerosis, to clarify why diabetes increases the risk and severity of atherosclerosis. Genetically engineered pig models are also increasingly used in single‐disease research, demonstrating their utility in simulating human disease onset and progression for conditions like cardiometabolic diseases, neurodegenerative disorders, and cancers.^73^, ^74^, ^75^ However, their application in modeling multiple co‐occurring diseases remains less common. Future research should introduce them into multimorbidity studies by introducing multiple pathogenic genes or combining induction methods to reveal complex disease interactions. However, formidable husbandry costs and long generation times restrict their use in preclinical validation.

### 4.5 Non‐human primates (NHPs)

NHPs represent the gold standard for biological fidelity in preclinical research. They naturally develop a spectrum of age‐associated diseases and closely replicate human pathologies. Different primate species offer distinct advantages for modeling human aging‐related diseases. For neurological studies, gray mouse lemurs model cognitive decline, while common marmosets offer insights into neurodegeneration via spontaneous Aβ/tau aggregates.^76^, ^77^ For systemic aging research, chimpanzees develop human‐like metabolic syndrome, CVD, and renal dysfunction,^78^ and rhesus macaques replicate a wide spectrum of age‐related physiological and pathological changes.^79^ Primates are also essential for studying pathogenesis, transmission, and vaccine development. Many human viruses fail to replicate in mice or induce distinct pathologies, whereas NHPs develop human‐like traits, making them crucial for viral infection research.

While the research potential of NHPs is invaluable due to their similarity to humans, this potential is offset by stringent ethical standards and higher economic costs. Consequently, their application in multimorbidity research should be strategically prioritized for areas with the highest translational potential and where no alternative models suffice.

## 5 FUTURE PERSPECTIVES

Our historical understanding of multimorbidity primarily relies on common shared histopathological hallmarks across co‐occurring diseases, yet we are still uncertain about the cell types or signaling pathways that drive multimorbidity progression.^80^ Although multiple laboratory animal models have been developed across species in multimorbidity research, the key issue is that no single model is sufficient to mimic the complicated multimorbidities found in humans. Invertebrates like *Drosophila* and *C. elegans* are excellent for decoding conserved disease mechanisms for age‐related conditions.^81^ Rodent models, enhanced by advanced gene‐editing technologies, are nearly ubiquitous in human disease modeling. However, the phenotypic differences with humans often lead to translational failures. Companion animals, like cats and dogs, which share human environments, are key for studying environmental influences. Large animals, such as pigs and NHPs, offering more human‐like traits, are critical for metabolic, neurological, and infectious diseases research. However, their use is limited by cost, ethical constraints, and gene‐editing challenges. Each model offers unique value yet poses specific limitations. To overcome the current limitations and authentically capture the complexity of multimorbidity, future research should focus on the following strategic areas:

First, a cross‐species validation platform should be implemented, leveraging the unique strengths of different models: simple organism for initial high‐throughput genetic and drug screening → rodents for in‐depth mechanistic analysis → large mammals for final preclinical validation and systemic pathophysiology.

Second, there is an urgent need to standardize multimorbidity models. Combined models should be developed that incorporate various risk factors including biological (aging, gender, obesity), behavioral (diet, alcohol, drugs, smoking), and psychological (depression, anxiety) elements, and integrate various methods (induced, spontaneous, surgical, genetically engineered). Standardized protocols should be established covering genetic background, gender, age, and housing conditions. Comprehensive phenotyping criteria should be developed across physiology, biochemistry, histopathology, etiology, and immunology. Scientific evaluation standards should be implemented to guarantee model reliability and reproducibility.

Third, advanced human‐cell‐based models—specifically, human‐derived, 3D micro‐cultured tissues (organoids) and microfluidic circuits that emulate organ‐level physiology (organ‐on‐a‐chip, OOC)—enable multimorbidity modeling by integrating patient‐specific tissues within a dynamic micro‐system.^82^, ^83^ These technologies can be employed for high‐throughput mechanistic screening and disease‐specific interaction studies in a human genetic context, presenting a powerful in vitro approach. For the future, a promising synergistic pipeline should involve validating findings from these human in vitro systems in animal models to integrate the strengths of reductionist approaches with the systemic insight of whole‐body physiology.

Lastly, ethical oversight must evolve in tandem with model complexity. Refining induction methods to minimize animal suffering, implementing humane endpoint criteria, and establishing clear ethical standards for the use of higher‐order species are paramount.

## 6 CONCLUSION

Multimorbidity presents a formidable challenge to global health and biomedical research, and leads to declining function, reduced quality of life, increased premature mortality and a high economic burden. The reason for the co‐occurrence of multiple conditions within an individual involve complex, interrelated biological processes, such as aging, genetic factors, chronic inflammation, metabolic dysregulation, insulin resistance, immune dysfunction, persistent infections, and mental disorders, which interact through shared physiological pathways. These intricate interactions present major obstacles to elucidating the mechanisms of multimorbidity. This review has cataloged clinical patterns of multimorbidity and animal platforms (as shown in Table 1), revealing a critical gap: we lack models that capture the dynamic reciprocity between diseases. To address this, we emphasize the need to diversify animal models—beyond commonly used inbred mouse strains—and expand modeling strategies (including genetic editing, environmental induction, surgical intervention, and combinatorial approaches) to better mimic the complex clinical pathology of multimorbidity at the whole‐organism level. As the burden of multimorbidity continues to rise, a more integrated research strategy is urgently needed. Combining the strengths of in vitro (such as orgnoid and OoC) and in vivo systems will be crucial to decode the complex pathophysiological networks of co‐occurring diseases and translate these insights into improved clinical outcomes.

**TABLE 1:** Summary of animal models in multimorbidity research.

Species	Applications	Key advantages	Major limitations
*C. elegans*	**Age‐related multimorbidities:** Metabolic disorders: obesity and diabetes^84^, ^85^ Neurodegenerative diseases: AD,^86^ PD,^87^ ALS^88^	**High‐throughput screening** due to short lifespan, ease of cultivation, and low cost. **Powerful genetic tools** and fully sequenced genome facilitate mechanistic studies of conserved pathways.	**Low physiological translatability** to humans due to lack of advanced organs (adaptive immune system, complex brain). **Cannot model systemic, multi‐organ interactions** central to human multimorbidity.
Mice	**Cardiometabolic:** DMs with CVD,^89^ NAFLD,^90^ and Cancers^91^; CVD with cancers,^92^ depression,^93^ and Cognitive impairment,^94^ CKD^95^ **Infectious:** HIV with TB,^96^ CVD,^97^ depression^98^; TB with DMs^99^; COVID‐19 with CVD, DMs^100^ **Psychiatric‐Physical:** Alzheimer's dementia with depression and anxiety^101^, ^102^; Anxiety, Depression with CVD, cancers^55^, ^103^	**Unparalleled genetic toolbox** (GEM, CRIPSR) for modeling specific disease interactions. **Low cost and abundant strains** enable large‐scale, hypothesis‐driven studies. Collaborative Cross (CC) populations model **genetic diversity**.^56^, ^57^	**Significant physiological disparities** (metabolism, immune function, lifespan) often lead to poor translational outcomes. **Limited behavioral complexity** constrains modeling of advanced psychiatric multimorbidity. **Often require aggressive induction** (multiple mutations, severe stressors) not reflective of human disease progression.
Rat	**Cardiometabolic:** Hypertension, stroke, HF, metabolic disorder with cognitive impairment^104^, ^105^, ^106^; Stroke, MI with depression and anxiety^107^, ^108^ **Psychiatric‐Physical:** Chronic pain with depression/anxiety^109^; Epilepsy with depression,^110^ SCI with obesity^111^ and CVD and locomotor dysfunction^112^; HIV with neurocognitive disorders^113^ **Physical–physical:** Obesity and hypertension^114^; Diabetes with tumorigenesis and metastasis,^115^ infective endocarditis^116^; CKD with MI,^117^ mineral and bone disorder^118^; HBV, and HCV infection with HCC^119^	**Superior physiological & metabolic similarity** to humans (vs. mice), especially in cardiovascular and renal systems. **More complex and translatable behaviors** for neuropsychiatric and cognitive studies. **Pharmacokinetic profiles** are closer to humans.	**Higher cost and lower availability** of genetically modified models compared to mice. **Gene editing remains less established** than in mice, limiting rapid model generation. **Still exhibits fundamental species differences** from humans.
Golden hamster	**Infectious‐Physical:** Age‐related COVID‐19 severity^120^; COVID‐19 with obesity, diabetes^121^, ^122^ **Cardiometabolic:** Atherosclerosis with diabetes^123^ **Psychiatric‐Physical:** Stress‐induced obesity^55^	**Unique host susceptibility** to human respiratory viruses (such as SARS‐CoV‐2) with clinical‐like pathology. **Human‐like metabolic responses** to diet and stress, making them valuable for cardiometabolic comorbidity studies.	**Limited genetic and reagent toolbox** (antibodies, inbred strains) hinders mechanistic dissection. **Lack of standardized protocols** for multimorbidity induction and phenotyping. **Relatively underutilized**, leading to a smaller knowledge base.
Degus	**Age‐related multimorbidity:** Spontaneous development of AD‐like pathology (Aβ, NFTs) and T2D^62^, ^124^	**A rare natural model** for spontaneous, age‐related neurodegenerative and metabolic conditions, avoiding aggressive genetic or surgical induction.	**Extremely limited accessibility** and lack of standardized, characterized laboratory strains. **Scarce commercial reagents** and established protocols. **Long natural lifespan** can slow research progress
Dogs	**Age‐related multimorbidity:** Longitudinal cohorts (GRLS, DAP) study cancer, CKD, cognitive decline co‐occurrence^68^, ^125^ **Psychiatric‐Physical:** Epilepsy with anxiety/depression^126^; Attention‐deficit hyperactivity disorder (ADHD) with compulsive behavior, aggressiveness and fearfulness^127^	**Shares human environment and aging processes**, providing unparalleled insight into environment–gene‐disease interactions. **Complex behavioral repertoire** allows for robust assessment of neuropsychiatric traits. **Phenotypic diversity** mirrors human population heterogeneity.	**High cost and logistical challenges** of maintaining large cohorts. **Limited ability to control genetic and environmental variables** compared to laboratory species. **Ethical and regulatory considerations** for experimental interventions.
Pigs	**Cardiometabolic:** DM, hypercholesterolemia, and CKD with coronary dysfunction^128^, ^129^; Heart failure with AD^130^ **Psychiatric‐Psychiatric:**Chronic pain with insomnia^131^	**Striking anatomical, physiological, and metabolic similarity** to humans, ideal for translational preclinical validation. **Advanced genetic engineering** is feasible and produces highly human‐relevant phenotypes. **Suitable size** for repeated sampling and use of clinical medical devices	**Very high husbandry costs and space requirements**. **Long gestation and maturation periods** slow model generation. **Fewer established, readily available multimorbidity models** compared to rodents
NHPs	**Age‐related multimorbidity:** Gray mouse lemur: cognitive impairment^76^ Chimpanzees: metabolic disorders and CVD,^132^ cardio‐renal dysfunction,^78^ and HPA dysregulation^132^ Rhesus macaques: age‐related physical^133^, ^134^ and psychological disorders^135^ **Infectious‐Physical:** HIV‐associated CVDs,^136^ gastrointestinal and liver dysfunction,^137^, ^138^ metabolic disorders^139^	**Closest phylogenetic relation to humans**, providing the highest fidelity for complex pathophysiology, immunology, and behavior. **Naturally develop a spectrum of age‐related chronic diseases**. **Essential for studying pathogenesis and therapies** where host specificity is critical (HIV).	**Extremely high cost, stringent ethical oversight, and specialized facilities** limit widespread use. **Long lifespans** make chronic studies challenging. **Low reproductive rate and limited genetic tools** hinder the creation of specific genetic models.

## AUTHOR CONTRIBUTIONS

**Xinpei Wang:** Conceptualization; software; visualization; writing – original draft; writing – review and editing. **Yakun Ren:** Investigation; validation; writing – original draft; writing – review and editing. **Xingjiu Yang:** Investigation; writing – review and editing. **Mengyuan Li:** Investigation; writing – review and editing. **Junxiu Liu:** Conceptualization; writing – review and editing. **Xiaoyan Du:** Conceptualization. **Wen Wang:** Writing – review and editing. **Ran Gao:** Conceptualization; funding acquisition; supervision; validation; writing – review and editing.

## FUNDING INFORMATION

This work was supported by the Chinese Academy of Medical Sciences Innovation Fund for Medical Sciences (2023‐I2M‐2‐001), the State Key Laboratory Special Fund (2060204), the Non‐profit Central Research Institute Fund of Chinese Academy of Medical Sciences (2023‐PT180‐01), the Young Elite Scientists Sponsorship Program of China Association for Science and Technology (grant 2020QNRC001).

## CONFLICT OF INTEREST STATEMENT

The authors declare no competing interests. Ran Gao is an editorial board member of *AMEM* and a corresponding author of this article. To minimize bias, she was excluded from all editorial decision making related to the acceptance of this article for publication.

## ETHICS STATEMENT

Not applicable.
